# Hierarchical-Structured Fe_2_O_3_ Anode with Exposed (001) Facet for Enhanced Lithium Storage Performance

**DOI:** 10.3390/nano13132025

**Published:** 2023-07-07

**Authors:** Yanfei Liu, Jianfei Lei, Ying Chen, Chenming Liang, Jing Ni

**Affiliations:** 1 Longmen Laboratory, School of Physics and Engineering, Henan University of Science and Technology, Luoyang 471000, China; 2 School of Chemistry and Material Science, Hubei Engineering University, Xiaogan 432000, China

**Keywords:** doping, surface technology, lithium-ion battery, energy storage, iron oxide

## Abstract

The hierarchical structure is an ideal nanostructure for conversion-type anodes with drastic volume expansion. Here, we demonstrate a tin-doping strategy for constructing Fe_2_O_3_ brushes, in which nanowires with exposed (001) facets are stacked into the hierarchical structure. Thanks to the tin-doping, the conductivity of the Sn-doped Fe_2_O_3_ has been improved greatly. Moreover, the volume changes of the Sn-doped Fe_2_O_3_ anodes can be limited to ~4% vertical expansion and ~13% horizontal expansion, thus resulting in high-rate performance and long-life stability due to the exposed (001) facet and the unique hierarchical structure. As a result, it delivers a high reversible lithium storage capacity of 580 mAh/g at a current density of 0.2C (0.2 A/g), and excellent rate performance of above 400 mAh/g even at a high current density of 2C (2 A/g) over 500 cycles, which is much higher than most of the reported transition metal oxide anodes. This doping strategy and the unique hierarchical structures bring inspiration for nanostructure design of functional materials in energy storage.

## 1. Introduction

With the development of portable electronics and electric vehicles, advanced lithium-ion batteries (LIBs) have become urgently desirable [1,2,3,4,5]. The key issue for high performance batteries (high energy/power density) is developing advanced anode materials with high specific capacity and high electronic/ionic conductivity. Anode materials play an important role in LIBs. The property of anode, such as the specific capacity, stability, conductivity, and diffusion of Li^+^ ions, is associated with the excellent performance of the batteries. Graphite was the earliest anode material used in the commercial lithium-ion batteries. In the development process of lithium-ion batteries, graphite plays a crucial role, and it continues to play an irreplaceable role in the anode material of present energy storage batteries [6]. The traditional graphite anode has a lower specific capacity (only 372 mAh/g) and poor diffusion of Li^+^ ions, which cannot be satisfied with the development of the advanced batteries. The lithium metal anode has been widely studied due to its high specific capacity (3860 mAh/g), low redox potential (−3.04 V vs. SHE), and low weight density (0.534 g/cm). The uneven deposition of lithium metal can induce the severe lithium dendritic growth and the drastic volume changes, leading to the low Coulombic efficiency and potential safety hazards [7]. In recent years, strategies such as improving solid-state electrolyte interface (SEI) films [8], constructing artificial layers [9], and modifying diaphragms [10] have been proposed to stabilize the issues with the lithium metal anode. However, the stability and safety have not been well solved, restricting the commercial application of lithium metal. Si-based anodes (mainly Si and SiO*_x_*) also have very high theoretical specific capacity (4200 mAh/g), while the significant volume exchanges during the charging/discharging cycles lead to the continuous destruction of the electrode materials and the repeated break of SEI films, leading to a serious capacity fading [11]. Transition metal oxides (TMOs) have attracted extensive attention recently due to their high theoretical specific capacities, good safety performance, and their cheap and easy preparation [12]. In 2000, researchers first proposed that TMOs should be used as negative electrodes for lithium batteries [13]. Since then, research on TMO materials has been increasing and TMOs have become one of the important candidate materials in the field of anode materials. Actually, TMO anodes have the same problems as Si-based anodes. For example, the cyclic stability and the rate capacity of TMO anodes are not very good because of the volume effects and the low charge diffusion. Among numerous TMOs, Fe_2_O_3_ has been regarded as one of the most promising candidates for advanced LIBs because of its high theoretical capacity (1005 mAh/g), which is ~2.7 times greater than that of commercial graphite (372 mAh/g) [14,15,16,17,18]. However, the increased capacity based on its conversion-type lithium storage is generally accompanied by many challenges, such as low electron conductivity, low ion diffusion, and huge volume change [19,20,21]. The conductivity and the stability of Fe_2_O_3_ electrodes are equally important to the high performance of batteries, which means that the excellent electron/ion transport can contribute to the high-rate performance, and the super stable electrode can contribute to the long-cycling performance [22,23,24,25,26,27,28,29]. In contrast, the poor conductivity of electrodes will prevent electrons and ions diffusing rapidly in the solid bulk, which causes the serious polarization and the uneven lithification of the Fe_2_O_3_ electrode. Finally, the power density and the safety of batteries will be problems due to the poor conductivity of the material [30,31]. Additionally, large volume changes of Fe_2_O_3_ anodes during the lithification/delithification processes will lead to the collapse of electrode structure, thus leading to the destruction of the solid electrolyte interface (SEI) films [32,33,34]. Therefore, constructing well-formed Fe_2_O_3_ anodes with fast ionelectron diffusion and with accommodation for volumetric expansion are guarantees for high performances of batteries.

Many efforts have been made to improve the conductivity and the stability of Fe_2_O_3_ anodes, and many efficient strategies, such as reducing size, conductive agent coating, conductive component composition, heterogeneous element doping, and novel nanostructure designs, have been proposed to tackle the problems associated with the low rate capability and the poor cyclic stability [30,35,36,37,38]. Recent studies have proved that the crystal planes and the hierarchical structures of electrode materials have significant effects on the electrochemical properties. For example, it has been confirmed that the energy barrier for Li-ion transfer across the (001) facet of Fe_2_O_3_ is much lower than that across the (110) facet [39]. Again, the gradient-structured Fe_3_O_4_/C nanospheres can effectively relieve the stress concentration caused by drastic volume changes and can derive an excellent stability during fast charging/discharging processes [40]. Therefore, studies on the controllable synthesis of the given crystal planes and on the design of the unique structures for Fe_2_O_3_ materials are of great interest. Many reports indicate that element doping can tune the facets of Fe_2_O_3_, especially for the exposed (001) facet tuning of Fe_2_O_3_ [7,17,36]. Based on the understanding of the literature, the Sn-doped Fe_2_O_3_ samples can more easily expose the (001) facet than pure Fe_2_O_3_ due to the Sn-doping. Moreover, when Sn atoms are introduced into Fe_2_O_3_, an impurity state can be induced in the bandgap of pure Fe_2_O_3_, resulting in the improved conductivity of Sn-doped Fe_2_O_3_. In addition, the Sn doping can induce the formation of belts with an exposed (001) facet, which can be stacked into ordered hierarchical structures [38,39]. Thus, we use a tin-doping strategy here to easily fabricate Fe_2_O_3_ material with the dual structural features of an exposed (001) facet and hierarchical structures. The exposed (001) facet provides channels for rapid Li^+^ ion diffusion, and the hierarchical structures accommodate the large volume expansion.

## 2. Materials and Methods

### 2.1. Materials Synthesis

Sn-doped Fe_2_O_3_ samples were prepared using an improved hydrothermal reaction [41]. The typical procedure is as follows: 150 mL 0.5 mol/L FeCl_3_ aqueous solution and 50 mL 0.012 mol/L K_2_SnO_3_ aqueous solution were prepared, respectively. Here, for the convenience of expression, one solution containing Fe^3+^ is indicated by Fe-sol and another solution containing SnO_3_^2−^ is indicated by Sn-sol. Firstly, the Sn-sol was added dropwise to the Fe-sol while stirring vigorously for 30 min to obtain a clear mixed solution. Then, the mixed system was incubated at 180 °C for 5 h in a Teflon-lined autoclave and cooled down to room temperature after the hydrothermal reaction. Finally, the products were collected through a succession of processes including centrifuging, washing, and drying. Pure Fe_2_O_3_ samples were also prepared using the same process of the Sn-doped Fe_2_O_3_ samples except for the absence of SnO_3_^2−^ ions for comparison.

### 2.2. Structure Characterization

The morphologies of the samples were obtained using a field emission scanning electron microscope (SEM, JSM-5610LV, Tokyo, Japan), and the electron diffraction spectrum was performed on a field emission transmission electron microscope (TEM, FEI Talos F200X, Hillsborough, NC, USA). The energy dispersive spectrum and the elemental mappings of the samples were tested using X-ray photoelectron spectroscopy (XPS, Thermo ESCALAB 250Xi, Waltham, MA, USA). The crystallographic information for the samples was investigated with X-ray powder diffraction (XRD, Bruker D8 Advance, Karlsruhe, Germany).

### 2.3. Electrochemical Tests

The electrochemical measurements were carried out by using CR 2025 coin-type cells. The working electrode was prepared using an electrode slurry on a copper current collector (11 µm thick). The slurry was composed of active material (Sn-doped Fe_2_O_3_), carbon (Super-P-Li), and polymer binder (sodium carboxymethyl cellulose, CMC, 3%) with a weight ratio of 8:1.5:0.5. The total mass loaded on each electrode was about 1.5 mg. Lithium wafer was used as both the counter electrode and the reference electrode. The cell was assembled in a high-purity argon filled glove box. A microporous polypropylene membrane (Cellgard 2300, Charlotte, NC, USA) was used as the separator, and the electrolyte was LiPF_6_ (1.0 mol/L) in a 1:1 (*w*/*w*) mixture of ethylene carbonate (EC) and dimethyl carbonate (DMC). Charge/discharge tests were performed using a Land testing system (CT2001A, China) at different current densities with voltage windows of 0.05–3 V (vs. Li_+_/Li) and the electrochemical data were obtained using an electrochemical workstation (CHI 650E, China).

### 2.4. Calculation

The DFT calculations were carried out by Vienna Ab initio Simulation Package (VASP) [42]. Core electrons were described using pseudopotentials generated from the projector augmented wave method [43], and valence electrons were expanded in a plane-wave basis set with an energy cutoff of 440 eV. The Perdew–Burke–Ernzerh (PBE) exchange correlation functional with the on-site Coulomb Repulsion U term was used for Fe_2_O_3_. The value of U was selected to be 4.0 eV for Fe atoms. The Fe_2_O_3_ (001) surface was simulated using a slab model with a (1 × 1) Fe_2_O_3_ unit. A vacuum layer of 16 Å along with *Z*-axis direction was applied to avoid interaction between images caused by periodic boundary conditions. The Sn-doped Fe_2_O_3_ was constructed by replacing one Fe atom with Sn atom in the Fe_2_O_3_ cell. The convergence criteria of force and energy were set as 0.01 eV/Å and 10^−4^ eV, respectively. The surface free energies of the Fe_2_O_3_ (001) surface and Sn-doped Fe_2_O_3_ (001) surface were calculated using the following equation:

$$E_{surf}=\frac{E_{slab}-nE_{bulk}}{2S}$$

where *E_bulk_* is the per formula unit energy of bulk Fe_2_O_3_ (Sn-doped Fe_2_O_3_), *E_slab_* is the energy of the surface, and *S* is the area of the surface.

## 3. Results and Discussion

**Figure 1:**
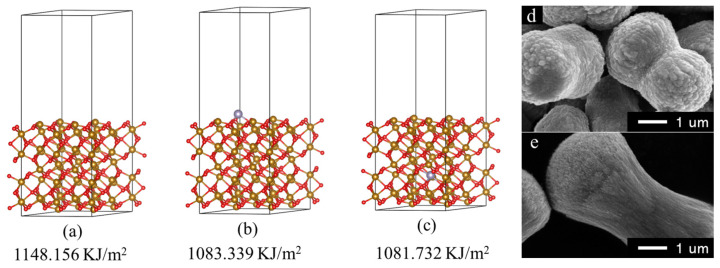
Optimized structures of the (001) surface and corresponding surface energy: pure Fe_2_O_3_ (**a**) and Sn-doped Fe_2_O_3_ (Sn(wt%) = 6.0% (**b**) surface doping; (**c**) internal doping) and the great effect on the morphologies of Fe_2_O_3_ by ultra-trace tin element ((**d**) pure Fe_2_O_3_, (**e**) Sn-doped Fe_2_O_3_).

Figure 1 shows the optimized structures of (001) facets and the corresponding surface energy of Fe_2_O_3_ and Sn-doped Fe_2_O_3_. The Sn-doped Fe_2_O_3_ was constructed by replacing one Fe atom with a Sn atom in the Fe_2_O_3_ cell, and the Sn-doping concentration is set as 6% (wt%) to be consistent with the experimental values. Compared to pure Fe_2_O_3_ (Figure 1a), the surface energy of the Sn-doped Fe_2_O_3_ (Figure 1b,c) is decreased by about 64 KJ/m^2^ (surface doping) and 66 KJ/m^2^ (internal doping), respectively. Clearly, the Sn-doping can reduce the surface energy of the (001) facet greatly. The calculation results indicate that a small amount of Sn-doping in Fe_2_O_3_ can easily make the (001) facet exposed. Based on this understanding, we prepared Sn-doped Fe_2_O_3_ powders experimentally with a low Sn-doping concentration used as anode materials with the (001) facet exposed for LIBs. Pure Fe_2_O_3_ powders were also prepared using the same process as the Sn-doped Fe_2_O_3_ powders for comparison to investigate the further effects of the Sn-doping on the microstructures. As shown in Figure 1d,e, two samples have similar morphologies to a peanut-like contour but a significant difference in the secondary structure. Clearly, pure Fe_2_O_3_ samples are dense aggregates formed by the accumulation of nanoparticles, while Sn-doped Fe_2_O_3_ samples are loose stacks formed by one-dimensional (1D) nanowires. For electrode materials with volume expansion, the hierarchical structures with 1D wires and 3D stack brushes should be ideal structures for accommodating the expanded volume and releasing stress. Therefore, the electrode structure will be relatively stable during the charging/discharging processes compared to the dense structures. Moreover, the loose structure is conducive to the contact between the electrolyte and the active material, and the diffusion rate of Li^+^ ions will be faster in loose structures than that in dense structures, hence the better rate performance of electrode materials.

**Figure 2:**
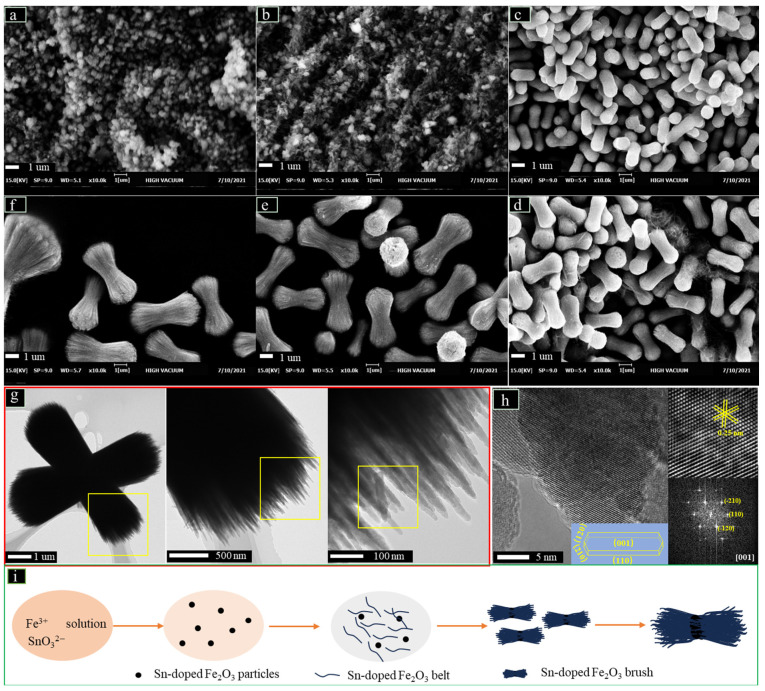
Time-dependent structures of Sn-doped Fe_2_O_3_ ((**a**) 0.5 h, (**b**) 1 h, (**c**) 2 h, (**d**) 3 h, (**e**) 5 h, (**f**) 7 h). TEM images of Sn-doped Fe_2_O_3_ (**g**). HRTEM images together with FFT patterns (**h**). Insets: drawing of a belt and the formation diagram of stacked peanut-like brushes (**i**).

Figure 2 investigates the effects of the reaction time on the morphologies of the Sn-doped samples, thereby revealing the formation process of stacked peanut-like brushes. It can be seen from Figure 2a that only particles are present at the initial stage of the reaction (0.5 h). However, when the reaction time reaches 1 h (Figure 2b), many filamentous materials appear around the particles, and they are gradually aggregated and eventually stacked together to form peanut-like hierarchical structures (Figure 2c). The following processes are similar to Ostwald ripening (Figure 2d–f), in which the filamentous materials continue to stack on the original hierarchical structures forming larger particles. Therefore, the process of Sn-doped Fe_2_O_3_ brushes forming can be illustrated as in Figure 2i. Firstly, Sn-doped Fe_2_O_3_ nanoparticles are formed at the initial hydrothermal reaction. Then, many filaments appear around the nanoparticles and gather with each other. Finally, the excess nanobelts are aggregated and stacked orderly to form larger brushes. To represent the formation of stacked peanut-like brushes, the TEM images (Figure 2g) show that the stacked lines present ribbon-like structures. The HRTEM image and the FFT patterns (insets in Figure 2h) indicate that three sets of lattice fringes (0.25 nm) fit well to the α-Fe_2_O_3_ (110), (−120), and (−210) facets, respectively [9,26], indicating the basal plane of the nanowire is the (001) facet.

**Figure 3:**
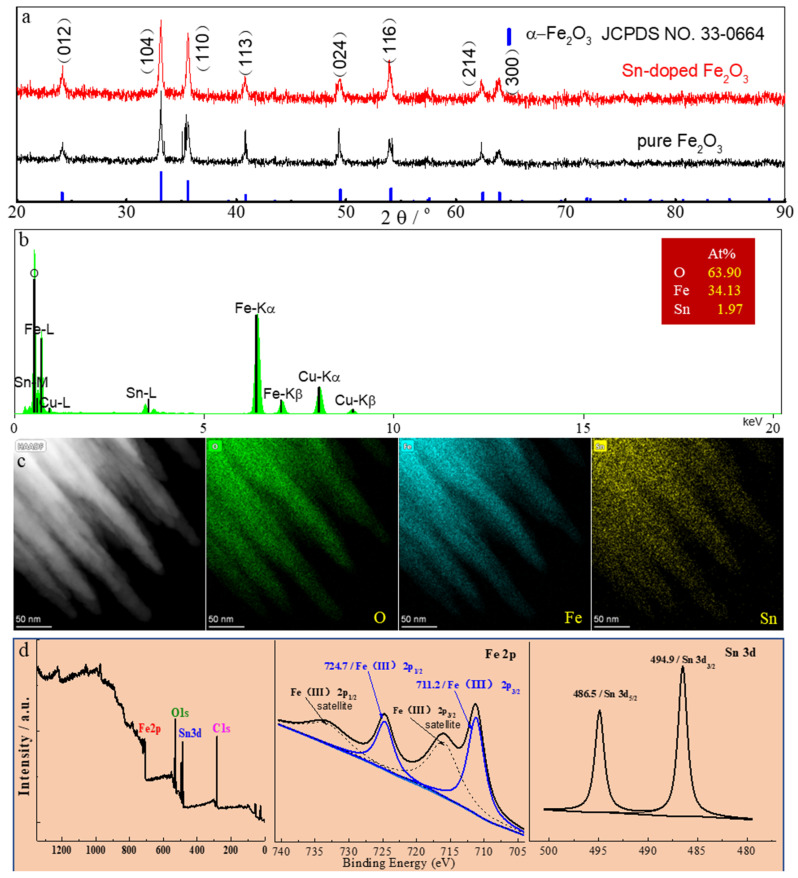
XRD patterns (**a**), EDS spectra (**b**), STEM-EDS elemental mapping (**c**), and XPS (**d**) of Sn-doped Fe_2_O_3_ samples.

Figure 3 shows results of the crystal structure, the component, and the elemental distribution of the Sn-doped Fe_2_O_3_. As shown in Figure 3a, XRD patterns of the prepared sample (Sn-doped Fe_2_O_3_) and the pure Fe_2_O_3_ sample are all perfectly matched with the standard JCPDS card no ^#^33-0644 of α-Fe_2_O_3_, and the peaks located at 24.1°, 33.2°, 35.6°, 40.8°, 49.5°, 54.1°, 62.5°, and 64.0° correspond to the (012), (104), (110), (113), (024), (116), (214), and (300) reflections of α-Fe_2_O_3_. Furthermore, the typical peaks of the Sn-based oxide cannot be observed in the Sn-doped Fe_2_O_3_ sample, revealing the doping of tin atoms as substitutes for iron atoms instead of forming new phases. The EDS spectrum (Figure 3b) provides the data of elements and their contents in the prepared sample. The mass percentage of the Sn element in the prepared sample is about 7.4%, which is close to the concentration of 6.0% for the optimized structures (Figure 1b,c). The average content of the Sn element in the Sn-doped Fe_2_O_3_ is 7.1%, which was detected using inductively coupled plasma mass spectrometry (ICP). The STEM-EDS elemental mappings in Figure 3c indicate that Fe, O, and Sn elements are uniformly distributed in the wires, which has also been confirmed by the XPS results in Figure 3d.

**Figure 4:**
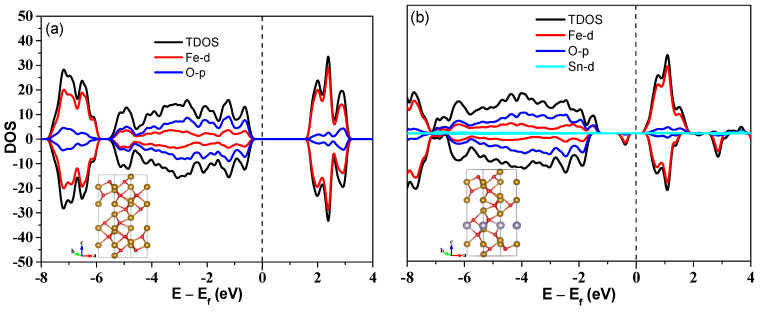
Density of states of Fe_2_O_3_ (**a**) and Sn-doped Fe_2_O_3_ (**b**). The fermi level is set to zero as shown by the dashed lines.

**Figure 5:**
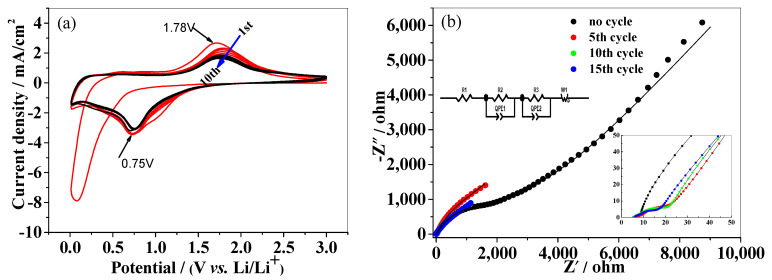
CV curves at a scanning rate of 2 mV/s (**a**) and EIS plots (**b**) of Sn-doped Fe_2_O_3_ anode.

Good conductivity is crucial to an electrode material for batteries. Many methods, such as element doping and carbon coating, can improve the conductivity of materials. Element doping to achieve bulk conductivity has become a popular method for preparation of the material. Based on theoretical calculations, we found that the Sn-doping not only tunes the surface morphology but also enhances the conductivity of Fe_2_O_3_. The density of states of pure Fe_2_O_3_ and Sn-doped Fe_2_O_3_ are shown in Figure 4. Clearly, pure Fe_2_O_3_ is an insulator with a bandgap of 2.16 eV. When Sn atoms are introduced into Fe_2_O_3_, an impurity state is induced in the bandgap of pure Fe_2_O_3_. The calculation results indicate that the Sn-doping can improve the conductivity of Fe_2_O_3_ indeed. The test results of powder conductivity also illustrate this conclusion. It can be seen from Table 1 that the resistivity values have decreased from the order of magnitude of 10^6^ to 10^4^ and the conductivity values have increased from the order of magnitude of 10^−7^ to 10^−5^, indicating a change by 2 orders of magnitude from pure Fe_2_O_3_ to Sn-doped Fe_2_O_3_. For anode materials of lithium-ion batteries, good conductivity is not only benefitial for the cycling stability, but also for the rate performances during the charging/discharging cycles. It is well known that the electrochemical performances are not only based on the intrinsic crystallinity, but also related to the morphologies and assembled structures of anode materials. To understand the enhancement of this hierarchical structure to the performance of lithium storage, the as-prepared Sn-doped Fe_2_O_3_ samples’ electrochemical performances as anode materials for LIBs are evaluated by using a two electrode coin-type cell. The electrochemical performances of the as-prepared Sn-doped Fe_2_O_3_ were investigated using cyclic voltammetry (CV) and electrochemical impedance spectroscopy (EIS) measurements. The CV measurement for Sn-doped Fe_2_O_3_ is shown in Figure 5a. In the first cathodic sweep, there is no typical reduction peak at ~0.8V for Fe^3+^ to Fe^0^ compared to the CV curves of the pure Fe_2_O_3_ (Figure S2), while a broad irreversible peak can be observed at the low potential (0–0.5V), indicating that the side reactions of the electrode material are significant. Additionally, this process should be accompanied by the reduction and irreversible decomposition of the electrolyte to form solid electrolyte interphase (SEI) films [44]. Furthermore, the reduction reactions from Fe_2_O_3_ to Fe^0^ as shown at below in the first cathodic sweep should occur.

Fe_2_O_3_ + 2Li^+^ + 2e^−^ → Li_2_(Fe_2_O_3_) (1)

Li_2_(Fe_2_O_3_) + 4Li^+^ + 4e^−^ → Fe^0^ + 3Li_2_O (2)

In the first anodic sweep, only a peak at 1.78 V can be observed, which is related to the oxidative reaction of Fe^0^ to Fe^3+^ as shown below.

2Fe^0^ + 3Li_2_O ↔ Fe_2_O_3_ + 6Li^+^ + 6e^−^ (3)

In the subsequent charging/discharging processes, the main cathodic peaks and anode peaks are slightly shifted, and the peak intensity decreases significantly. The results indicate the capacity loss and some irreversible processes occurred during the lithium ions insertion and extraction in the first cycle. It is noteworthy that no typical peaks of Sn lithification/delithification can be observed on the CV curves, meaning that there may be two possibilities: one is that the doping amount of Sn in Fe_2_O_3_ is too small to cause changes in the entire curves, and the other is that Sn atom does not participate in the lithium alloying reactions. Based on the literature, the lithium storage capacity contributed by the doped Sn element is very low due to the trace doping amount, which cannot be measured in the electrochemical testing curves [29]. Importantly, after the second cycle, both the CV peak positions and the integrated areas for Sn-doped Fe_2_O_3_ sample remain almost unchanged, while the overlap of the CV curves for the pure Fe_2_O_3_ in the first five cycles is not good (Figure S2), suggesting good capacity retention and structural stability of the Sn-doped Fe_2_O_3_ anode.

Figure 5b shows the Nyquist plots of EIS obtained from the Sn-doped Fe_2_O_3_ samples before cycling and after the 5th, the 10th, and the 15th cycle, respectively. The insert in Figure 5b is the equivalent circuit model and the partial enlargement of images of Nyquist plots. The corresponding fitted impedance parameters are listed in Table 2. In the circuit model, *R1* is the ohmic contact resistance of the inside battery, *R2* is the charge transfer impedance, *R3* is the resistance of the formed SEI films after cycles, and *W* is the Warburg impedance. It can be seen from Table 2 that the values of *R1* are very small in different charging and discharging states of batteries, indicating that the various components inside the battery are in good contact. The electrode materials are in an inactive state before the battery undergoes charging/discharging cycles, so the charge transfer resistance (*R2*) value of the materials is relatively larger, reaching 1135 Ω. After the battery has carried out several cycles, the electrode materials reach full activation, and correspondingly, the values of *R2* are decreased significantly and decreased to 112.6 Ω, 163.1 Ω, and 168.7 Ω after the 5th, the 10th, and the 15th cycle, respectively. It is noteworthy that the fitted value of *R3* before cycling is very small (almost negligible), while after charging/discharging cycles, it reaches more than 10 Ω. More importantly, after undergoing 5 cycles, 10 cycles, and 15 cycles, the values of *R3* are not changed significantly. Based on the results, it can be inferred that the surfaces of Sn-doped Fe_2_O_3_ have changed greatly due to the Li^+^ insertion/extraction and the formation of SEI films. Generally, it is inevitable that electrolyte is decomposed on the surface of anode materials with a lower lithium intercalation potential to form SEI films. Another valuable result obtained from the EIS experiment is that the values of *R2* and *W* have scarcely changed after the 5th, the 10th, and the 15th charging/discharging, meaning that the formation of the SEI films does not affect the reactions of Li^+^ ion insertion/extraction in Sn-doped Fe_2_O_3_. Therefore, the good cycling stability and high Li^+^ ion storage of the Sn-doped Fe_2_O_3_ are mainly attributed to the (001) facet and the unique hierarchical structures of Sn-doped Fe_2_O_3_. These features are vital for the stability, the safe operation, and the rate capability of LIBs. The (001) facet has a shorter diffusion distance of lithium ions, which is beneficial to improving the rate performance, and the hierarchical structures can improve the stability and the specific capacity of Sn-doped Fe_2_O_3_ anode.

**Figure 6:**
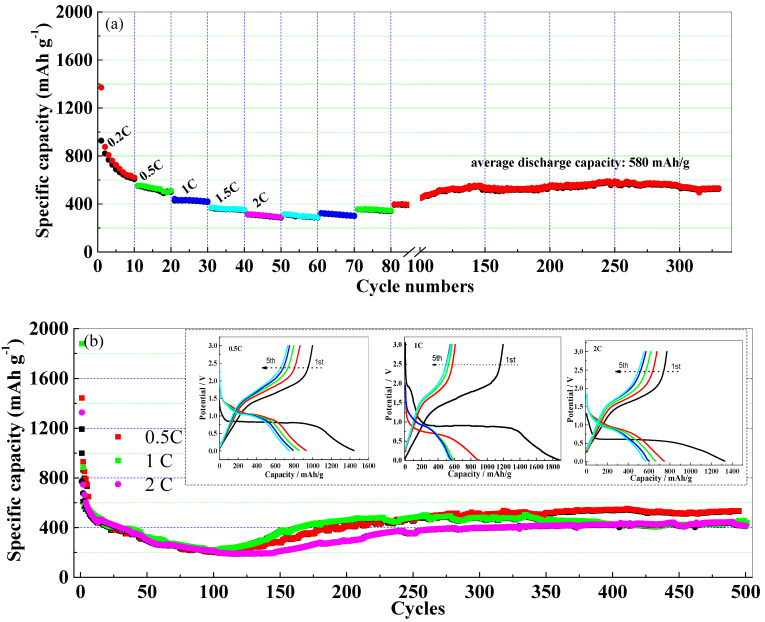
Rate performances at different charging/discharging current densities (**a**) and cycling stability of Sn-doped Fe_2_O_3_ anode at larger charging/discharging current densities (**b**).

Figure 6 shows the rate performances and the cycling performances of the Sn-doped Fe_2_O_3_ samples. As a comparison, the results of the electrochemical tests for pure Fe_2_O_3_ are shown in Figure S1. In the rate plots (Figure 6a), the average discharge capacities are about 779, 529, 431, 360, and 304 mAh/g at 0.2C, 0.5C, 1C, 1.5C, and 2C current densities, respectively. Except for a 32% reduction in capacity from 0.2C to 0.5C, in the following process, the capacity reduction is less than 20%, specifically, 19%, 16.5%, and 15.6% from 0.5C to 1C, 1C to 1.5C, and 1.5C to 2C, respectively. Compared with the pure Fe_2_O_3_ samples (Figure S1a), the improved rate performance for the Sn-doped Fe_2_O_3_ samples is attributed to its better conductivity and the rapid Li^+^ diffusion on the (001) facets [39]. The cycling stabilities of the Sn-doped Fe_2_O_3_ samples are also outstanding (Figure 6a,b). The average discharge capacity can be maintained at 580 mAh/g at 0.2 C, and it can still be maintained above 400 mAh/g even at a high current density (2C) after 500 cycles, which is much higher than that of the pure Fe_2_O_3_ at the same current density (Figure S1b). The advantage of our hierarchical structure is ensuring sufficient clearance and space, which can gradually relieve the stress concentration caused by the drastic volume changes. The hierarchical-structured brushes present a sustained buffering effect on the drastic volume changes of the active Fe_2_O_3_, thus retaining the completeness and the stability of the whole structure during the fast cyclic process (Figure S3). Moreover, the hierarchical structure gradually relies on the void spaces to alleviate the volume changes in the following discharging processes. About 4% of the vertical increase and 13% of the horizontal increase for the hierarchical-structured Sn-doped Fe_2_O_3_ brushes can be confirmed in comparison to the full charge/discharge states. The gram capacity is improved qualitatively, although a certain amount of volumetric energy density is sacrificed. Thus, the morphology of SEI layer and the whole electrode can be retained after hundreds of cycles, suggesting an ultra-long life of the hierarchical-structured Sn-doped Fe_2_O_3_ anodes.

## 4. Conclusions

In conclusion, Sn-doped Fe_2_O_3_ materials have been prepared using a hydrothermal method. The prepared Fe_2_O_3_ samples have hierarchical structures constructed by stacked nanowires with exposed (001) facets. When used as anodes for lithium-ion batteries, the Sn-doped Fe_2_O_3_ materials exhibit excellent rate performance and good cycling stability. Consequently, the hierarchical structures of the Sn-doped Fe_2_O_3_ could be one of the potentially promising candidates as anodes for the next generation of high-power lithium-ion batteries.

## Figures and Tables

**Table 1:** Resistivity and conductivity values of Fe_2_O_3_ and Sn-doped Fe_2_O_3_.

	Samples	1MPa	2MPa	3MPa
Resistivity/Ω·m	Fe_2_O_3_	2.01 × 10^6^	1.70 × 10^6^	1.53 × 10^6^
	Sn-doped Fe_2_O_3_	4.83 × 10^4^	3.47 × 10^4^	2.88 × 10^4^
Conductivity/S·m^−1^	Fe_2_O_3_	4.97 × 10^−7^	5.89 × 10^−7^	6.59 × 10^−7^
	Sn-doped Fe_2_O_3_	2.07 × 10^−5^	2.88 × 10^−5^	3.47 × 10^−5^

**Table 2:** Impedance parameters of the Sn-doped Fe_2_O_3_ electrodes before cycling and after the 5th, the 10th, and the 15th cycle of discharge/charge (*R1*, Ohm resistance; *R2*, charge-transfer resistance; *R3*, SEI film resistance; *W*, Warburg impedance).

	*R1* (Ω)	*R2* (Ω)	*R3* (Ω)	*W* (Ω)
Before cycling	8.44	1135	0.51	6.42
The 5th cycle	7.48	112.6	12.64	7.53
The 10th cycle	8.21	163.1	17.97	6.89
The 15th cycle	7.96	168.7	18.09	6.61

## Data Availability

Data sharing is not applicable to this article.

## Supplementary Materials

The following supporting information can be downloaded at https://www.mdpi.com/article/10.3390/nano13132025/s1, Figure S1: Rate performances at different charging/discharging current densities (a) and cycling stability of pure Fe_2_O_3_ anode at 0.2C current density (b); Figure S2: CV curves at a scanning rate of 2 mV/s of pure Fe_2_O_3_ anode; Figure S3: SEM images of Sn-doped Fe_2_O_3_ anodes before cycling (a) and after the 5th discharge cycle (b). Inset: the statistics of particle size distribution in both horizontal and vertical directions.
