# Supplementary material for: Hierarchical-Structured Fe2O3 Anode with Exposed (001) Facet for Enhanced Lithium Storage Performance
Source: Nanomaterials (Basel). 2023 Jul 7;13(13):2025. doi: 10.3390/nano13132025 (PMC10343585; doi:10.3390/nano13132025)
Supplement: Supplementary file 1 [file nanomaterials-13-02025-s001.zip › nanomaterials-2471090-supplementary.pdf]

Supporting Information

Hierarchical-structured Fe_2O_3 anode with exposed (001) facet for enhanced lithium storage performance

Yanfei Liu [1], Jianfei Lei [1,*], Ying Chen [1], Chenming Liang [1] and Jing Ni [2,*]

[1] School of Physics and Engineering, Longmen laboratory, Henan University of Science and Technology, Luoyang 471000, China
[2] School of Chemistry and Material Science, Hubei Engineering University, Xiaogan 432000, Hubei, China
* Correspondence: leijianfei9966@163.com (J.L.); JingNi@hbeu.edu.cn (J.N.)

Figures

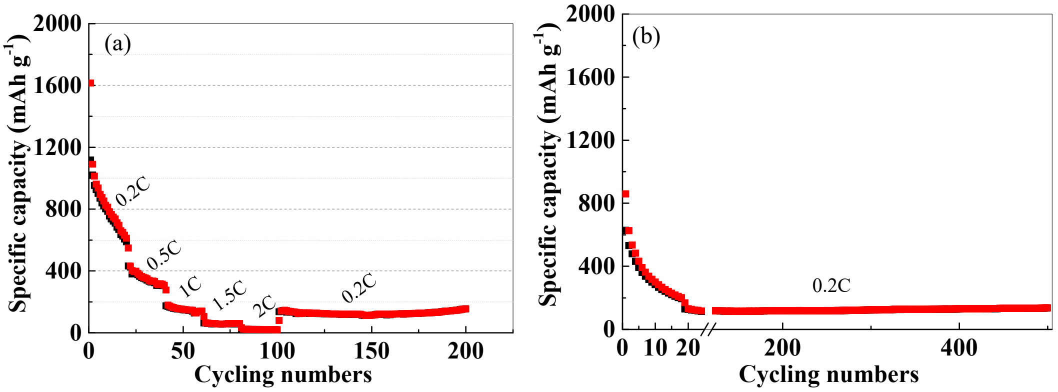

Figure S1 Rate performances at different charging/discharging current densities (a) and cycling stability of pure Fe_2O_3 anode at 0.2C current density (b).

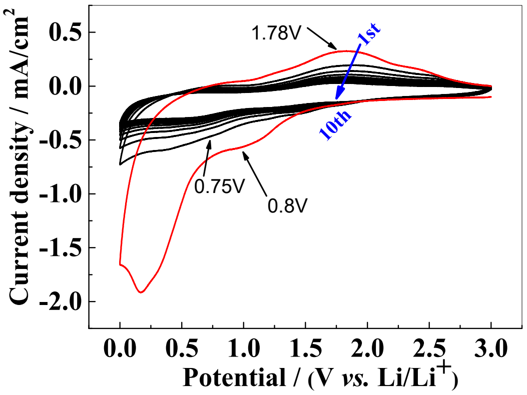

Figure S2 CV curves at a scanning rate of 2mV/s of pure Fe_2O_3 anode.

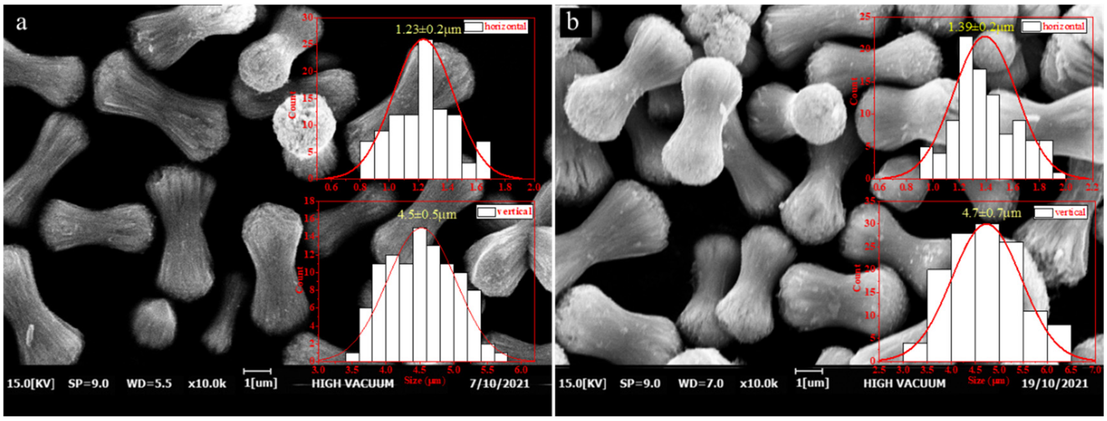

Figure S3 SEM images of Sn-doped Fe_2O_3 anode before cycle (a) and after the 5th discharge cycle (b). inset: the statistics of particle size distribution in both horizontal and vertical directions.
